# Revisiting the strategies for the pharmacological management of type 2 diabetes – From glycemic control, organ protection, safety to weight reduction

**DOI:** 10.1111/jdi.13726

**Published:** 2021-12-14

**Authors:** Hung‐Yuan Li

**Affiliations:** ^1^ Division of Endocrinology and Metabolism Department of Internal Medicine National Taiwan University Hospital Taipei Taiwan

## Abstract

With the development of several new classes of anti‐diabetic drugs in the past decade, the concept for the pharmacological management of type 2 diabetes is evolving from ‘glycemic control’ to ‘organ protection’. Besides, the increase in the prevalence of an aging population urges a strategy focusing on safety and de‐intensification. In the coming years, with the development of several novel and potent anti‐obesity drugs, the ‘weight‐centric’ strategy is expected to gain more and more attention in the treatment of diabetes, since it can both prevent diabetic complications and optimize the quality of life. With all these strategies, clinicians should consider the patient’s clinical characteristics and make an individualized goal through shared decision making with the patient.

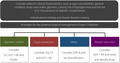

The goals of diabetes care are to prevent complications and to optimize the quality of life. Based on these goals, several strategies have been developed and are still evolving for the pharmacological management of type 2 diabetes. In the past decade, various new anti‐diabetic drugs have been developed, including dipeptidyl peptidase‐4 (DPP‐4) inhibitors, sodium‐glucose cotransporter 2 (SGLT2) inhibitors, glucagon‐like peptide‐1 receptor agonists (GLP‐1 RAs), and several insulin analogs. The findings from cardiovascular and renal outcome trials of SGLT2 inhibitors and GLP‐1 RA have led to a paradigm shift in the treatment of type 2 diabetes. In addition to glycemic control, cardiovascular or renal protective effects beyond glycemic control, or the ‘organ protection’ effects, have been emphasized in several guidelines published by academic associations. On the other hand, with the global trend of an aging population, a different strategy should be considered to treat diabetic patients of advanced age, focusing primarily on safety issues. Recently, the development of novel anti‐obesity drugs with clinically significant weight reduction effects has shed some light on a new ‘weight‐centric’ strategy for the treatment of diabetes. Therefore, it is time to revisit different strategies in the pharmacological management of type 2 diabetes.

Findings from several cardiovascular or renal outcome trials of SGLT2 inhibitors and GLP‐1 RAs have demonstrated their ‘organ protection’ effects in people with type 2 diabetes. As a result, the guidelines from academic associations recommend that physicians consider the baseline hemoglobin A1c (HbA1c) level independently and prescribe SGLT2 inhibitors or GLP‐1 RAs with proven cardiovascular or renal benefits to diabetic patients at risk of or with established atherosclerotic cardiovascular diseases, heart failure, or diabetic kidney diseases. However, the results from meta‐regression of the cardiovascular outcome trials have demonstrated that there is a significant association between HbA1c reduction and the risk of major adverse cardiovascular events, especially for the risk of stroke^1^. These findings restate the importance of glycemic control in reducing cardiovascular events in diabetic patients. It is well known that the United Kingdom Prospective Diabetes Study (UKPDS) and the UKPDS follow‐up study showed the relationship between intensive glycemic control and a reduced risk of microvascular and macrovascular complications and all‐cause mortality^2^. In a meta‐analysis of data in the UKPDS, the Action to Control Cardiovascular Risk in Diabetes (ACCORD) trial, the Action in Diabetes and Vascular Disease: Preterax and Diamicron MR Controlled Evaluation (ADVANCE) trial, and the Veterans Affairs Diabetes Trial (VADT), a 0.88% decrease in HbA1c was associated with a 15% reduction in the risk of fatal and non‐fatal myocardial infarction^3^, which is comparable to the ‘organ protection’ effects demonstrated in the cardiovascular outcome trials. Therefore, in diabetic patients with poor glycemic control or a high HbA1c, anti‐diabetic drugs with a potent glucose‐lowering effect, such as sulfonylurea, some GLP‐1 RAs, and insulin, should be considered preferentially.

The ‘organ protection’ and the ‘glycemic control’ strategies emphasize more the prevention of complications. In contrast, in diabetic patients of old age, with fragility, terminal diseases, or disability, optimizing the quality of life should be the focus in the treatment of diabetes. Therefore, avoiding the side effects of anti‐diabetic medications is a major consideration in choosing appropriate drugs for these patients. These patients have some common clinical features, including decreased appetite, cognitive impairment, functional disability, being vulnerable to fall and fracture, and reduced renal function etc. As a result, they more easily develop hypoglycemia and are less likely to respond appropriately. Hypoglycemia is associated with an increased risk of arrhythmia, and diabetic microvascular and macrovascular complications. Besides, hypoglycemia may impair cognitive function and forms a vicious cycle together with fragility and dementia. Thus, de‐intensification or the withdrawal of medications with a risk of hypoglycemia should be considered, such as sulfonylurea and insulin. In addition, the incidence of side effects of certain anti‐diabetic medications in addition to hypoglycemia may have unfavorable effects on the quality of life, such as gastrointestinal side effects for GLP‐1 RAs, genital or urinary tract infection for SGLT2 inhibitors, and edema for thiozolidinediones. On the other hand, adverse events of DPP‐4 inhibitors are less frequent, which make DPP‐4 inhibitors the preferred choice in these patients. Taken together, in diabetic patients of old age, with fragility, terminal diseases, or disability, DPP‐4 inhibitors should be prioritized for their safety among the other anti‐diabetic medications, and de‐intensification should be considered.

The ‘weight‐centric’ strategy has been proposed for a long time and is back in focus recently with the development of novel and potent drugs for weight reduction^4^. Obesity is an important pathophysiology of diabetes and its complications. Besides, obesity also leads to the development of various risk factors of atherosclerosis, such as hypertension and dyslipidemia. Therefore, focusing on weight reduction to improve glycemic control and to prevent complications is a reasonable approach for the management of diabetes. Indeed, weight reduction by more than 5% is beneficial for the management of diabetes, hypertension, and dyslipidemia, and medications for these diseases usually can be reduced. Weight reduction by more than 10% can have an additional benefit for obesity‐related diseases, such as non‐alcoholic fatty liver disease, sleep apnea, cardiovascular diseases, or even reducing cardiovascular mortality. In addition, weight reduction can also improve fitness and may have psychological and social benefits. However, there have only been limited therapeutic options in previous years. Lifestyle modification has been shown to be effective. Nonetheless, a team‐based approach and coaching are usually needed for its success, and it is often difficult to maintain sustained weight loss in the long term. On the other hand, bariatric surgery is another effective option, but it is limited because of its short‐term and long‐term adverse effects. There have been some drugs for weight reduction previously. Orlistat is associated with only a modest weight‐reducing effect, which limits its benefits in the management of diabetes and diabetic complications. Phentermine is another drug for weight reduction. However, it is only approved for short‐term use, mainly in the USA, and is associated with adverse events in the cardiovascular, gastrointestinal, and central nervous systems. Among anti‐diabetic drugs, SGLT2 inhibitors, GLP‐1 RAs, and metformin are associated with weight loss, but most of these drugs have only a modest effect in weight reduction. Therefore, the beneficial effects of weight reduction on metabolic control or on reducing cardiovascular risks by these drugs are usually not expected. Recently, the GLP‐1 RA semaglutide, at a dose of 1.0 mg every week, was shown to have a more pronounced effect on weight reduction (3.5–5%). In the STEP 2 study, semaglutide 2.4 mg weekly was associated with an even greater effect on weight loss (6.2%), which was accompanied by a HbA1c decrease of 1.6% and the proportion of subjects achieving a HbA1c of <6.5% was 68%^5^. Similarly, liraglutide 3 mg per day was shown to have a greater effect on weight loss, compared with liraglutide at 0.6–1.8 mg per day (the doses for anti‐diabetic effects). In the future, there are several novel weight reduction drugs in the pipeline, such as the GLP‐1 and gastric inhibitory polypeptide (GIP) dual agonist tirzepatide, the amylin agonist cagrilintide, or the combination of semaglutide and cagrilintide. Based on current reports, these drugs were associated with a mean weight loss of 10.8–17.1%, and the proportion of subjects reaching a HbA1c of <6.5% was more than 80%. With the development of these new drugs, the ‘weight‐centric’ strategy aiming at a weight loss of more than 5–10% is emerging, and the selection of an appropriate population for this strategy as well as the individualized target and rate for weight reduction should be issues to be explored in the near future.

Figure 1 shows the proposed strategies for the pharmacological management of diabetes. To decide which strategy is to be used, clinicians should consider the patient’s clinical characteristics individually, such as age, comorbidities, general condition, body mass index, glycemic control, risk of hypoglycemia, and the risk of or the presence of diabetic complications. Then, clinicians should discuss with the patient and go through a shared‐decision‐making process. Once the strategy has been chosen, the indicated drugs shown in Figure 1 should be considered preferentially.

**Figure 1:**
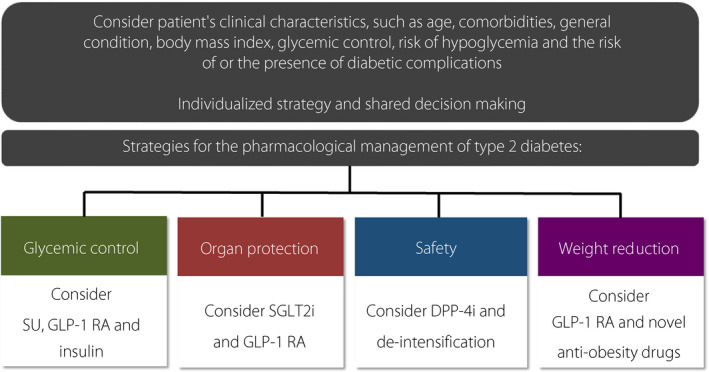
Proposed strategies for the pharmacological management of diabetes. DPP‐4i, dipeptidyl peptidase‐4 inhibitor; GLP‐1 RA, glucagon‐like peptide‐1 receptor agonist; SGLT2i, sodium‐glucose cotransporter 2 inhibitor; SU, sulfonylurea.

In conclusion, with the development of several new classes of anti‐diabetic drugs in the past decade, the concept for the pharmacological management of type 2 diabetes evolves from ‘glycemic control’ to ‘organ protection’. Besides, the increase in the prevalence of an aging population warrants a strategy focusing on safety and de‐intensification. In the coming years, with the development of several novel and potent anti‐obesity drugs, the ‘weight‐centric’ strategy is expected to gain more and more attention in the treatment of diabetes, since it can both prevent diabetic complications and optimize the quality of life. With all these strategies, clinicians should consider a patient’s clinical characteristics and make an individualized goal through shared decision making with the patient.
